# Intermittent Fasting Inhibits High-Fat Diet–Induced Atherosclerosis by Ameliorating Hypercholesterolemia and Reducing Monocyte Chemoattraction

**DOI:** 10.3389/fphar.2021.719750

**Published:** 2021-09-30

**Authors:** Yuanli Chen, Jiamin Su, Yali Yan, Qian Zhao, Jialing Ma, Mengmeng Zhu, Xiaoyu He, Baotong Zhang, Hongmei Xu, Xiaoxiao Yang, Yajun Duan, Jihong Han

**Affiliations:** ^1^ Key Laboratory of Metabolism and Regulation for Major Diseases of Anhui Higher Education Institutes, Hefei University of Technology, Hefei, China; ^2^ Department of Human Cell Biology and Genetics, Southern University of Science and Technology School of Medicine, Shenzhen, China; ^3^ College of Life Sciences, Key Laboratory of Medicinal Chemical Biology, Key Laboratory of Bioactive Materials of Ministry of Education, Nankai University, Tianjin, China

**Keywords:** intermittent fasting, atherosclerosis, monocyte, CCL2, cholesterol

## Abstract

Atherosclerosis is a major pathology for cardiovascular diseases (CVDs). Clinically, the intermittent fasting (IF) has been observed to reduce the risk of CVDs. However, the effect of IF on the development of atherosclerosis has not been fully elucidated. Herein, we determined the protection of IF against high-fat diet–induced atherosclerosis in pro-atherogenic low-density lipoprotein receptor deficient (LDLR^-/-^) mice and the potentially involved mechanisms. The LDLR^-/-^ mice were scheduled intermittent fasting cycles of 3-day HFD feeding *ad libitum* and 1 day fasting, while the mice in the control group were continuously fed HFD. The treatment was lasted for 7 weeks (∼12 cycles) or 14 weeks (∼24 cycles). Associated with the reduced total HFD intake, IF substantially reduced lesions in the *en face* aorta and aortic root sinus. It also increased plaque stability by increasing the smooth muscle cell (SMC)/collagen content and fibrotic cap thickness while reducing macrophage accumulation and necrotic core areas. Mechanistically, IF reduced serum total and LDL cholesterol levels by inhibiting cholesterol synthesis in the liver. Meanwhile, HFD-induced hepatic lipid accumulation was attenuated by IF. Interestingly, circulating Ly6C^high^ monocytes but not T cells and serum c-c motif chemokine ligand 2 levels were significantly reduced by IF. Functionally, adhesion of monocytes to the aortic endothelium was decreased by IF via inhibiting VCAM-1 and ICAM-1 expression. Taken together, our study indicates that IF reduces atherosclerosis in LDLR^-/-^ mice by reducing monocyte chemoattraction/adhesion and ameliorating hypercholesterolemia and suggests its potential application for atherosclerosis treatment.

## Introduction

Cardiovascular diseases (CVDs) are one of the leading causes of morbidity and mortality worldwide. It causes about 18.6 million annual deaths, which accounts for almost one-third of all deaths (Virani et al., 2020). Although the primary and secondary preventive strategies have significantly lowered the incidence of CVDs (Patel et al., 2018), the therapeutics are still unsatisfactory due to the complex and multifactorial etiology of CVDs. Atherosclerosis, which is caused by lipid/cholesterol metabolic disorders and chronic inflammation, is the major pathology for CVDs. Multiple factors can affect the onset, progression, and rupture of atherosclerotic plaques. Hypercholesterolemia, particularly the increased low-density lipoprotein cholesterol (LDL-C) level, is the major one of these risk factors (Yusuf et al., 2004).

At the initial stage of atherosclerosis, the excess LDL in circulation accumulates in the inner layer of artery (Gistera and Hansson, 2017), followed by multiple modifications. The modified LDL, such as oxidatively modified LDL (oxLDL), is pro-inflammatory and immunogenic (Libby et al., 2019). Thus, the oxLDL can activate endothelial cells (ECs) to express adhesion molecules and chemokines, thereby attracting monocytes to adhere and infiltrate into the underneath of the endothelium layer, where the cells can differentiate into macrophages (Libby, 2002; Gimbrone and García-Cardeña, 2013; Čejková et al., 2016). Macrophages can take up oxLDL and further differentiate into lipid-laden macrophage/foam cells, the main component of atherosclerotic lesions (Ley et al., 2011). Foam cells within plaques ultimately undergo apoptosis and necrosis. Necrotic cores formed at the advanced stage of atherosclerosis can arouse inflammation, which further exacerbates plaque vulnerability (Tabas and Bornfeldt, 2016; Gonzalez and Trigatti, 2017). Meanwhile, vascular smooth muscle cells (VSMCs) de-differentiate and migrate from the media to the intima (Basatemur et al., 2019), where they synthesize and release extracellular matrix, such as collagen and elastin, to stabilize atherosclerotic plaques (Hansson and Libby, 2006; Di Gregoli et al., 2017).

Clinically, lowering LDL is the common and effective method to prevent CVDs. Statins can potently reduce total and LDL cholesterol levels by reducing liver cholesterol synthesis through inactivation of 3-hydroxy-3-methylglutaryl-CoA reductase (HMGCR), the rate-limiting enzyme in cholesterol synthesis, and by enhancing LDL catabolism through the activation of hepatic LDL receptor (LDLR) expression. Although statins have been recommended as the first-line therapy for CVDs treatment (Jukema et al., 2012), the effect of statins on CVDs is limited. Moreover, the adverse effect on cognition, liver, and muscle by statins have been observed (Ridker et al., 2008; Reiner, 2014; Stroes et al., 2015; Thompson et al., 2016; Adhyaru and Jacobson, 2018; Williams et al., 2019). In fact, the chronic inflammation is another important risk factor for CVDs. Therefore, the anti-inflammatory intervention is capable of improving outcomes in patients with LDL-lowering therapy.

Intermittent fasting (IF) is an important non-pharmaceutical intervention to benefit individuals with overweight/obesity. Three kinds of IF methods, alternate-day fasting, modified fasting regimens, and time-restricted feeding (TRF), have been applied to humans and rodent models to reduce body weight (Patterson and Sears, 2017). However, their effect on glucose-regulatory markers, serum lipid profiles, and inflammation are controversial. The alternate-day fasting method consists of fasting days with no calories consumed and equal feeding days with foods and beverages consumed *ad libitum*. This alternate-day fasting method can improve lipid profiles and inflammation. However, fasting can cause extreme hunger and distraction with lower mood, which makes this method an unfeasible intervention for public health (Heilbronn et al., 2005; Appleton and Baker, 2015). The typical intermittent fasting or TRF (16 h fasting and 8 h eating) normally does not reduce energy intake but helps maintain fitness and ameliorates metabolic syndromes (Rynders et al., 2019). Bhoumik *et al* demonstrated that the TRF did not reduce serum triglyceride and cholesterol levels in rats, while only reducing triglyceride levels in humans (Bhoumik et al., 2020; Moon et al., 2020). Moreover, the TRF has no effect on HFD-induced inflammatory cytokines in mice (Delahaye et al., 2018). Modified fasting regimens, also called intermittent energy restriction, based on the popular 5:2 diet, consists of two nonconsecutive days of fasting and 5 days of *ad libitum* eating per week. The modified fasting regimens have been demonstrated to reduce body weight, improve lipid profiles, and reduce inflammation, while increasing self-confidence and positive mood in many studies (Johnson et al., 2007; Varady et al., 2013; Hoddy et al., 2016).

In addition to reduction of obesity and diabetes mellitus, studies also suggest fasting may improve chronic inflammatory diseases. Jordan *et al* determined that fasting reduces monocyte mobilization from bone marrow by activating the AMP-activated protein kinase and inhibiting systemic c-c motif chemokine ligand 2 (CCL2) production (Jordan et al., 2019). CCL2 is the main cytokine that attracts monocyte migration and infiltration to the inflamed organs (Zhu et al., 2021). The chrono-pharmacological targeting of the CCL2 and its receptor c-c motif chemokine receptor 2 (CCR2) inhibits atherosclerosis in ApoE^-/-^ mice (Winter et al., 2018). Circulating monocytes, which include both inflammatory Ly6C^high^ and patrolling Ly6C^low^ monocytes, can infiltrate into the plaque area where they differentiate into M1 or M2 macrophages depending on the different microenvironment stimuli (Mills et al., 2000; Geissmann et al., 2010; Gratchev et al., 2012).

In spite of the beneficial effects of fasting, if the modified fasting regimens can influence the development of atherosclerosis remains incompletely elucidated. In this study, we selected a modified fasting regimen in which the pro-atherogenic LDLR-deficient (LDLR^-/-^) mice were fed HFD in cycles of 3-day HFD feeding and 1-day fasting, while mice in the control group were fed HFD continuously. After 7 or 14 weeks (∼12 and 24 cycles) of treatment, we compared atherosclerosis between the mice with HFD feeding *ad libitum* and intermittent fasting HFD feeding. We also attempted to unveil the underlying mechanisms.

## Materials and Methods

### Reagents

Rabbit anti-mouse CD3-FITC, CD4-APC, CD8a-PE, CD45-PE/Cyanine5, F4/80-APC, CD11b-PE, and Ly6C-FITC antibodies were purchased from BioLegend (San Diego, CA, United States). Mouse anti-CD68 monoclonal antibody was purchased from Santa Cruz (Oregon, United States). Rabbit anti-CCL2, intercellular adhesion molecule 1 (ICAM-1), and vascular cell adhesion molecule 1 (VCMA-1) polyclonal antibodies were purchased from Abclonal (Wuhan, China). Mouse anti–smooth muscle α-actin (SMA) monoclonal antibody and rabbit anti-collagen type III alpha 1 (COL3A1) polyclonal antibody were purchased from Affinity Biosciences (Cincinnati, OH, United States). Goat anti-mouse IgG-TRITC antibody was purchased from Sigma-Aldrich (St Louis, MO, United States). Rabbit anti-collagen type I alpha 1 (COL1A1) polyclonal antibody and goat anti-rabbit IgG (H + L)-TRITC antibody were purchased from Proteintech Group (Chicago, IL, United States). Rabbit anti-matrix metallopeptidase 2 (MMP2) polyclonal antibody was purchased from Bioss ANTIBODISS (Beijing, China). Rabbit anti-MMP9 polyclonal antibody was purchased from HUABIO (Hangzhou, China). Mouse CCL2 and interleukin 1β (IL-1β) ELISA kits were purchased from CUSABIO (Houston, United States). The mouse interferon γ (IFN-γ) ELISA kit was purchased from Thermo Fisher Scientific (Waltham, MA, United States). The triglyceride LabAssay kit was purchased from WAKO (Kanagawa Prefecture, Japan). The total cholesterol (TC) enzymatic determination kit was purchased from APPLYGEN (Beijing, China). HFD (20% fat, 1.25% cholesterol and 0.5% sodium cholate) was purchased from Medicience (Jiangsu, China).

### *In vivo* Experiment

The protocols for *in vivo* study with mice were approved by the Ethics Committee of Hefei University of Technology (No. HFUT20190609001) and conformed to the Guide for the Care and Use of Laboratory Animals published by the NIH. LDLR^-/-^ mice (male, ∼10 weeks old) were randomly divided into two groups (12/group). The mice in the control group were fed HFD *al libitum*, while the mice in the IF group were fed HFD *al libitum* in cycles of 3-day feeding and 1-day fasting. The food intake in each cycle was recorded. After seven or 14 weeks of treatment, the mice were anesthetized with isoflurane (2% isoflurane/0.2 L O_2_/min), followed by collection of blood samples. Mice were then euthanized by CO_2_, and aorta and liver samples were collected. Serum was prepared and used to determine the levels of TC, high-density lipoprotein cholesterol (HDL-C), LDL-C, and triglyceride (TG), and the activity of aspartate aminotransferase (AST) or alkaline phosphatase (ALP). Serum CCL2, IL-1β, and IFN-γ levels were determined by ELISA kits.

To determine the effect of IF on monocyte chemoattraction and adhesion to endothelium, LDLR^-/-^ mice (∼10 week old, male) were randomly divided into the control and IF groups (6/group) and fed HFD as described above for 2 weeks. All the mice were anesthetized and euthanized for collection of blood and aorta samples individually.

### Determination of Lesions by Oil Red O, HE, and Picrosirius Red Staining

Aortas were collected and separated carefully from other tissues. After fixation in 4% paraformaldehyde/PBS for 24 h, aortas were washed with PBS 3 times and then stained with 0.5% Oil Red O solution (Liu et al., 2018). Each entire aorta was dissected to assess *en face* lesions except for aortic root, which was used to prepare frozen cross section for determination of sinus lesions. Atherosclerotic lesions in the *en face* aorta and sinus lesions in the cross section of aortic root were calculated by two individuals who were blinded to the experimental design and each other’s results using a computer-assisted image analysis protocol, according to the guidelines for experimental atherosclerosis studies described in the American Heart Association statement (Daugherty et al., 2017). The *en face* lesions are expressed as mean percentages of lesion areas in the whole aorta, while sinus lesions as μm^2^/section.

The thickness of fibrotic caps and necrotic core areas in lesion areas were calculated after hematoxylin and eosin (HE) staining of aortic root cross sections. The collagen content was determined by Picrosirius red staining of the frozen cross sections of the aorta root (Liu et al., 2018). All images were captured with a Zeiss Axio Scope.A1 microscope (Baden-Württemberg, Germany).

### Immunofluorescent Staining

To determine the expression of CD68, SMA, COL1A1, COL3A1, MMP2, MMP9, CCL2, VCAM-1, and ICAM-1 in lesions, the aortic root cross sections were thawed at room temperature (RT) for 30 min, then placed in PBS for 30 min to remove the embedding reagent, followed by permeabilization with 0.5% Triton X-100 for 45 min. After blocking with 2% BSA at RT for 2 h, the sections were incubated with primary antibodies (CD68, COL1A1, COL3A1, MMP2, MMP9, CCL2, VCAM-1, or ICAM-1, 1:200; SMA, 1:500; and normal IgG, 1:200) overnight at 4°C. After aspirating the primary antibodies and washing three times with PBS, the sections were then incubated with a rhodamine-conjugated secondary antibody (1:1000) at RT for 1 h. All sections were then stained with DAPI solution for nuclei. After captured, the mean fluorescent intensity (MFI) of each image was calculated as described (Zhang et al., 2013).

### Determination of Hepatic Lipid Content

After weighing and taking a photograph, a piece of liver was removed and paraffin sections were prepared by a standard procedure. The morphology of the liver was determined by HE staining. To quantify TG content, a piece of liver (∼50 mg) was homogenized in ∼1.1 ml 1xPBS. A portion of the homogenate (100 μL) was saved for the determination of protein content which was used to normalize TG levels. 1 ml homogenate was then used to extract total lipids followed by TG quantitative analysis by a kit. Liver TC levels were determined by the assay kit purchased from ApplyGEN (Beijing, China) (Chen et al., 2015).

### Quantitative RT-PCR (qRT-PCR)

After treatment, RNA was extracted from the liver or aorta using Trizol reagent (ZOMANBIO, Beijing, China). cDNA was synthesized using the cDNA Synthesis Kit (Vazyme, Nanjing, China). Individual quantitative PCR was performed using SYBR Green PCR Master Mix (Vazyme, Nanjing, Jiangsu, China) and the primers with sequences listed in Table 1. Expression of HMGCR, HMGC synthase (HMGCS), sterol-responsive element binding protein 2 (SREBP2), ATP-binding cassette transporter A1 (ABCA1), ABCG1/5/8, scavenger receptor class B type I (SR-BI), cholesterol 7-α hydroxylase (CYP7A1), apolipoprotein B (apoB), and apoE mRNA in the liver and expression of COL1A1, COL3A1, MMP2, and MMP9 in the aorta were normalized by glyceraldehyde-3-phosphate dehydrogenase (GAPDH) mRNA in the corresponding samples.

**TABLE 1:** Sequences of primers for qRT-PCR.

Gene	Sense	Anti-sense
ABCA1	5′-CAA​TGT​CAA​GGT​GTG​GTT​CAA​T-3′	5′-GCT​GCT​GTT​TAG​TGA​GGT​TCA​A-3′
ABCG1	5′-GCC​TAC​TAC​CTG​GCA​AAG​ACC-3′	5′-GAA​CAG​CAC​AAA​ACG​CAC​AG-3′
ABCG5	5′-TTC​ACA​AAC​ACC​TCC​CCT​TC-3′	5′-GTG​CAT​CTT​AGG​CAG​CTC​AG-3′
ABCG8	5′-TCC​GAG​GAG​AAC​AAG​CTG​TC-3′	5′-CAC​TGG​TCA​TGG​CTG​AGA​AA-3′
HMGCS	5′-CGA​TGG​TGT​AGA​TGC​TGG​AAA​G-3′	5′-CAT​CAG​TTT​CTG​AAC​CAC​AGT-3′
HMGCR	5′-CCG​GCA​ACA​ACA​AGA​TCT​GTG-3′	5′-ATG​TAC​AGG​ATG​GCG​ATG​CA-3′
APOB	5′-ATG​TCT​GAT​TAC​CCC​CAG​CA-3′	5′-GGG​CTC​ACA​TTA​TTG​GCT​GT-3′
APOE	5′-CAT​TGC​TGA​CAG​GAT​GCC​TA-3′	5′-TGT​GTG​ACT​TGG​GAG​CTC​TG-3′
SR-BI	5′-GGC​TGC​TGT​TTG​CTG​CG-3′	5′-GCT​GCT​TGA​TGA​GGG​AGG​G-3′
SREBP2	5′-CTG​CAG​CCT​CAA​GTG​CAA​AG-3′	5′-CAG​TGT​GCC​ATT​GGC​TGT​CT-3′
CYP7A1	5′-TGA​TCC​TCT​GGG​CAT​CTC​AAG​CAA-3′	5′-AGC​TCT​TGG​CCA​GCA​CTC​TGT​AAT-3′
COL1A1	5′-GAC​GCC​ATC​AAG​GTC​TAC​TG-3′	5′-ACG​GGA​ATC​CAT​CGG​TCA-3′
COL3A1	5′-GGA​GCC​CCT​GGA​CTA​ATA​GG-3′	5′-ATC​CAT​CTT​TGC​CAT​CTT​CG-3′
MMP2	5′-GCC​TCA​TAC​ACA​GCG​TCA​ATC​TT-3′	5′-CGG​TTT​ATT​TGG​CGG​ACA​GT-3′
MMP9	5′-CGT​GTC​TGG​AGA​TTC​GAC​TTG​A-3′	5′-TGG​AAG​ATG​TCG​TGT​GAG​TTC​C-3′

### FACS Analysis

After treatment, mouse blood was collected in a test tube coated with the anticoagulant. Red blood cells were removed by incubating blood samples in a red blood cell lysis buffer (Biosharp) for 15 min on ice, followed by the addition of cold PBS and centrifugation for 10 min at ∼400 g at 4^o^C. About 10^5^ re-suspended cells in 100 μL were added with the corresponding antibody (CD3/CD4/CD8 or CD45/CD11b/Ly6C) and incubated for 30 min at RT with protection against light. After washing with PBS, the cells were re-suspended in PBS and subjected to FACS analysis.

### Determination of Monocyte Adhesion to Aortic Endothelium

THP-1 cells (a human monocytic cell line) were labeled by carboxyfluorescein succinimidyl ester (CFSE, 5 μM). Aortas collected from LDLR^-/-^ mice after 2 weeks of IF treatment were separated carefully from other tissues and prepared into the segment of arch aorta, thoracic aorta, and abdominal aorta. Each of them was cultured in complete DMEM medium separately. The CFSE-labeled THP-1 cells were then added to the aortic segment and co-incubated for 1 h. After washing with PBS, the adherent THP-1 cells to the aortic endothelium were captured and counted with ImageJ. The adherent THP-1 cells in the control group were defined as 1.

### Statistical Analysis

All experiments were repeated at least 3 times, and the representative results are presented. All values are represented as means ± SD except where indicated. FACS data were output by CytExpret (Beckman Coulter, Inc. Version 2.1.0.92). The raw data were initially conducted normal distribution analysis using the Shapiro–Wilk method followed by Levene’s test for the homogeneity of the variances. GraphPad Prism software (version 7.0, GraphPad Software, San Diego, CA) was used for unpaired Student’s t-test. The difference was considered significant if *p* < 0.05.

## Result

### Intermittent Fasting Inhibits Atherosclerosis Progression

**FIGURE 1:**
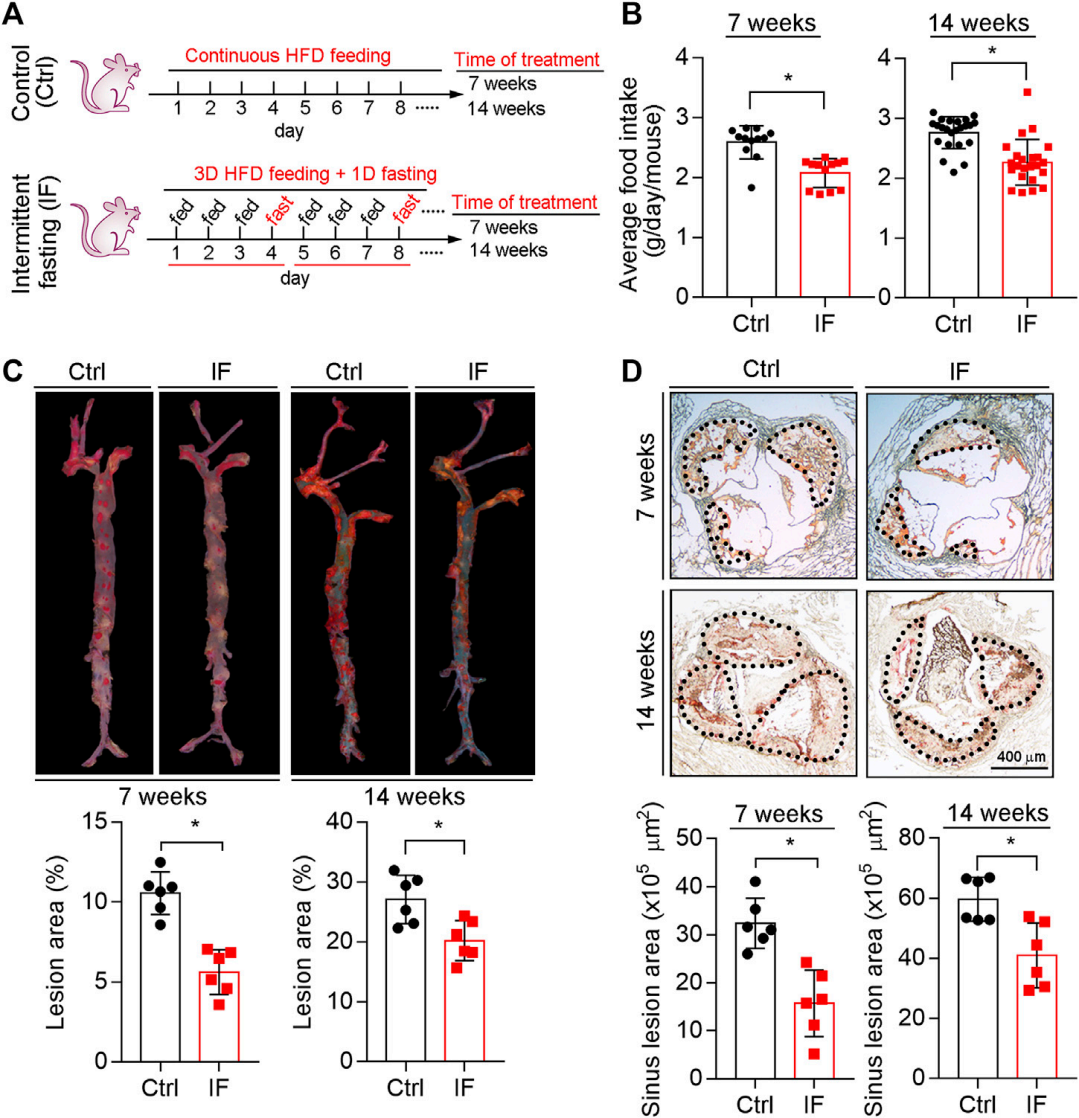
Inhibition of atherosclerosis in LDLR−/− mice by intermittent fasting treatment. **(A)** (experimental design): LDLR−/− mice were divided into four groups (*n* = 6), and received the following treatment for 7 or 14 weeks: Control groups, mice were fed HFD ad libitum; intermittent fasting (IF) groups, mice were fed HFD in cycles of 3-day HFD feeding and 1-day fasting. **(B)** The food intake in each cycle was determined and calculated as the daily average intake per mouse. *p < 0.05 [*n* = 12 (cycles) in 7 week groups, *n* = 23 (cycles) in 14 week groups]. **(C,D)** Lesions in the *en face* aortas **(C)** or sinus lesions in the aortic root **(D)** were determined by Oil Red O staining followed by quantitative analysis. Lesions in the en face aortas were expressed as a percentage of the whole aorta area, and sinus lesions were expressed as μm2. *p < 0.05, (*n* = 6). Ctrl: control; IF: intermittent fasting.

LDLR^-/-^ mice were scheduled continuous HFD feeding or cycles of a modified IF HFD feeding which consists of 3 days HFD feeding and 1 day fasting. The treatment lasted for 7 or 14 weeks (Figure 1A). We determined the food intake in each cycle and found the average food intake to be reduced by ∼20% by IF HFD feeding (Figure 1B).

At the end of the treatment, we collected mouse tissue samples including aortas. As shown in Figure 1C, compared with continuous HFD feeding, the *en face* lesions were reduced by 40 and 16% after 7 weeks and 14 weeks IF HFD feeding, respectively. We also determined the sinus lesions in aortic root were significantly reduced by ∼35 and 24% after 7 and 14 weeks IF, respectively (Figure 1D). Taken together, the data in Figure 1 indicate that IF can alleviate HFD-induced atherosclerosis.

### Intermittent Fasting Increases Plaque Stability

**FIGURE 2:**
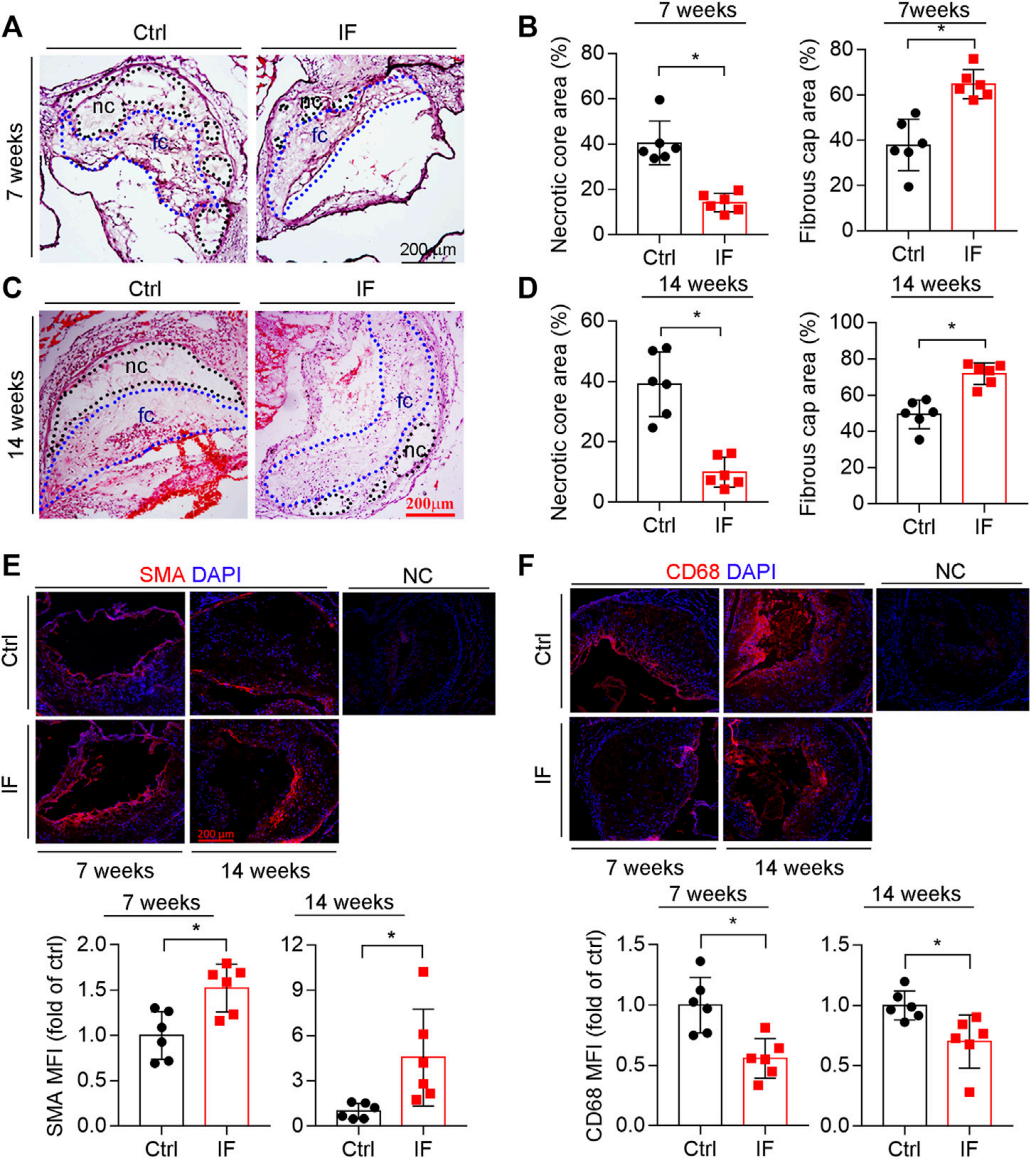
Intermittent fasting increases lesion plaque stability. The aortic root cross sections were used to complete the following assays: HE staining **(A,C)** with the quantitative analysis of necrotic core and fibrous cap areas **(B,D)**. nc: necrotic cores marked by a black dotted line; fc: fibrous cap marked by a blue dotted line. The expression of SMA and CD68 **(E,F)** in plaques were determined by immunofluorescent staining with the quantification of SMA- or CD68-positive areas. *p < 0.05 (n = 6).

The composition of plaques, such as collagen content, size of necrotic cores, and thickness of fibrous caps, can affect plaque vulnerability. The results of HE staining demonstrate that necrotic cores, which contain cholesterol crystal and apoptotic foam cells derived from macrophages and/or vascular smooth muscle cells (VSMCs), were reduced by IF (Figures 2A,C; left panels of 2B and D). In contrast, the thickness of fibrous caps (Figures 2A,C), which mainly consists of VSMCs and extracellular matrix to cover necrotic cores, was increased by the IF (right panels of Figures 2B,D).

VSMC is the major cell type in fibrotic caps and synthesizes/secretes extracellular matrix to stabilize lesions. We determined that SMA, a contractile VSMC marker, was elevated in the atherosclerotic plaques of the IF group (Figure 2E). Macrophage/foam cells were the main cell types within plaques and the component of necrotic cores. Macrophage/foam cells produce pro-inflammatory cytokines and matrix metalloproteinases (MMPs) to reduce lesion plaque stability. CD68 expression (the marker of macrophages) was substantially decreased by IF (Figure 2F).

**FIGURE 3:**
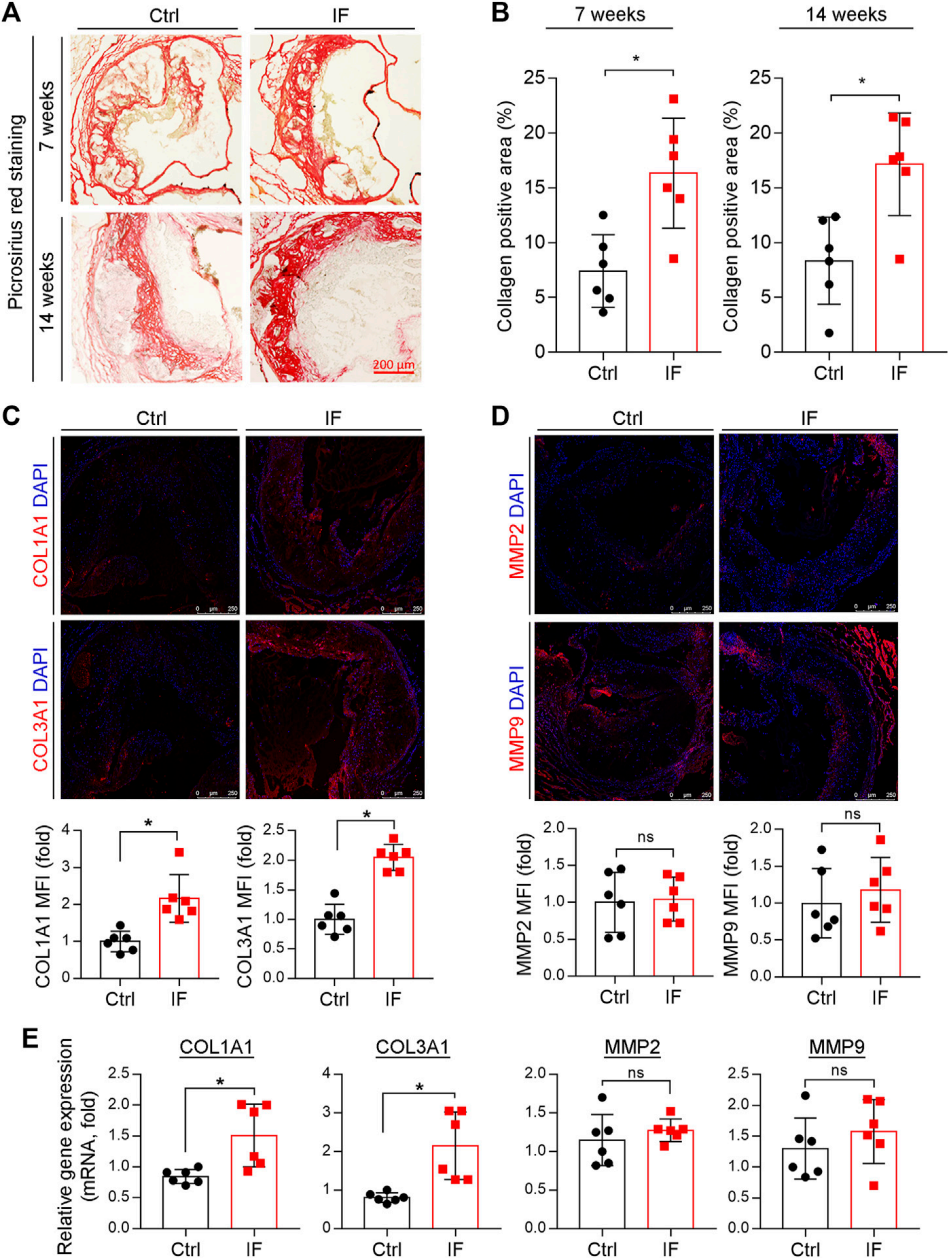
Intermittent fasting increases collagen content via inducing COL1A1 and COL3A1 expression. **(A,B)** The aortic root cross sections were used to determine collagen content by Picrosirius red staining with quantitative analysis of collagen positive areas. *p < 0.05 (*n* = 6). **(C,D)** The expression of COL1A1, COL3A1, MMP2, and MMP9 in plaques were determined by immunofluorescent staining with the quantification of positive staining areas. *p < 0.05, ns: not significant (*n* = 6). **E**: LDLR−/− mice were divided into the control or IF group (*n* = 6/group). After 2 weeks HFD feeding ad libitum or IF treatment, the mice aorta samples were collected. The expression of COL1A1, COL3A1, MMP2, and MMP9 were determined by qRT-PCR. *p < 0.05, ns: not significant (*n* = 6).

The increased positive area of Picrosirius red staining indicates that the collagen content in the arteries of mice in the IF group is more than the control group (Figures 3A,B). Indeed, the expression of collagen genes, COL1A1 and COL3A1, were increased by IF in the lesion areas and aortas (Figures 3C,E). However, the MMP2 and MMP9, enzymes to degrade extracellular matrix, were not affected by IF (Figures 3D,E).

Taken together, the results in Figures 2, 3 show that IF potentially increases plaque stability by increasing collagen and VSMC content, while decreasing necrotic cores and macrophage/foam cell content.

### Intermittent Fasting Lowers Cholesterol Levels by Inhibiting Liver Cholesterol Synthesis

**FIGURE 4:**
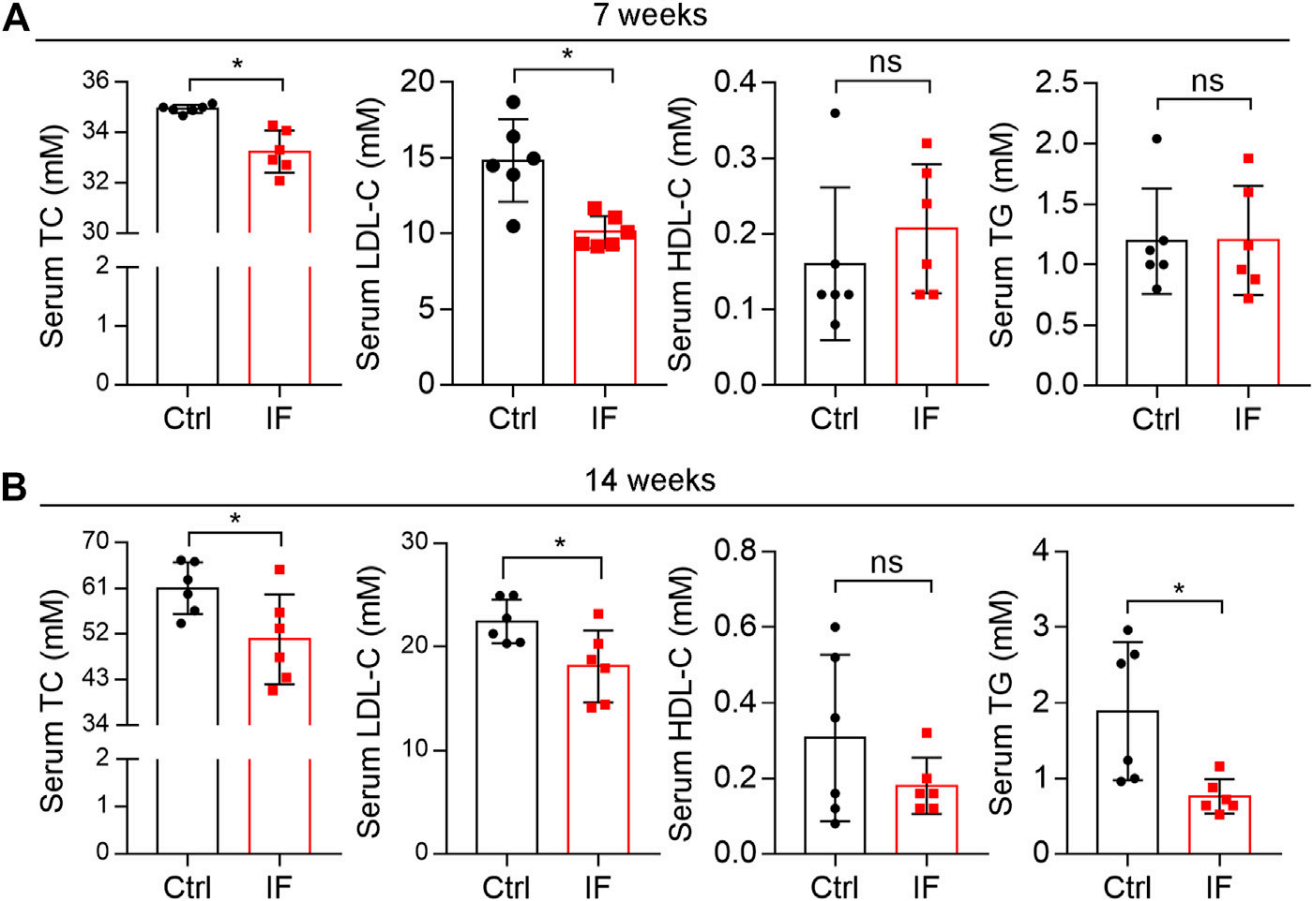
Intermittent fasting reduces hypercholesterolemia. Mouse serum samples isolated form LDLR-/- mice after 7 **(A)** or 14 weeks **(B)** IF feeding were used to detect levels of total cholesterol (TC), low-density lipoprotein cholesterol (LDL-C), high-density lipoprotein cholesterol (HDL-C), and triglyceride (TG) using an automatic biochemical analyzer. *p < 0.05, ns: not significant (*n* = 6). Ctrl: control; IF: intermittent fasting.

Serum TC and LDL-C levels are the most important risk factors for atherosclerosis. We determined both TC and LDL-C levels were significantly decreased in IF groups while HDL-C levels were not affected (Figure 4). Meanwhile, serum TG levels were also reduced in the 14 weeks IF group (right panel, Figure 4B).

**FIGURE 5:**
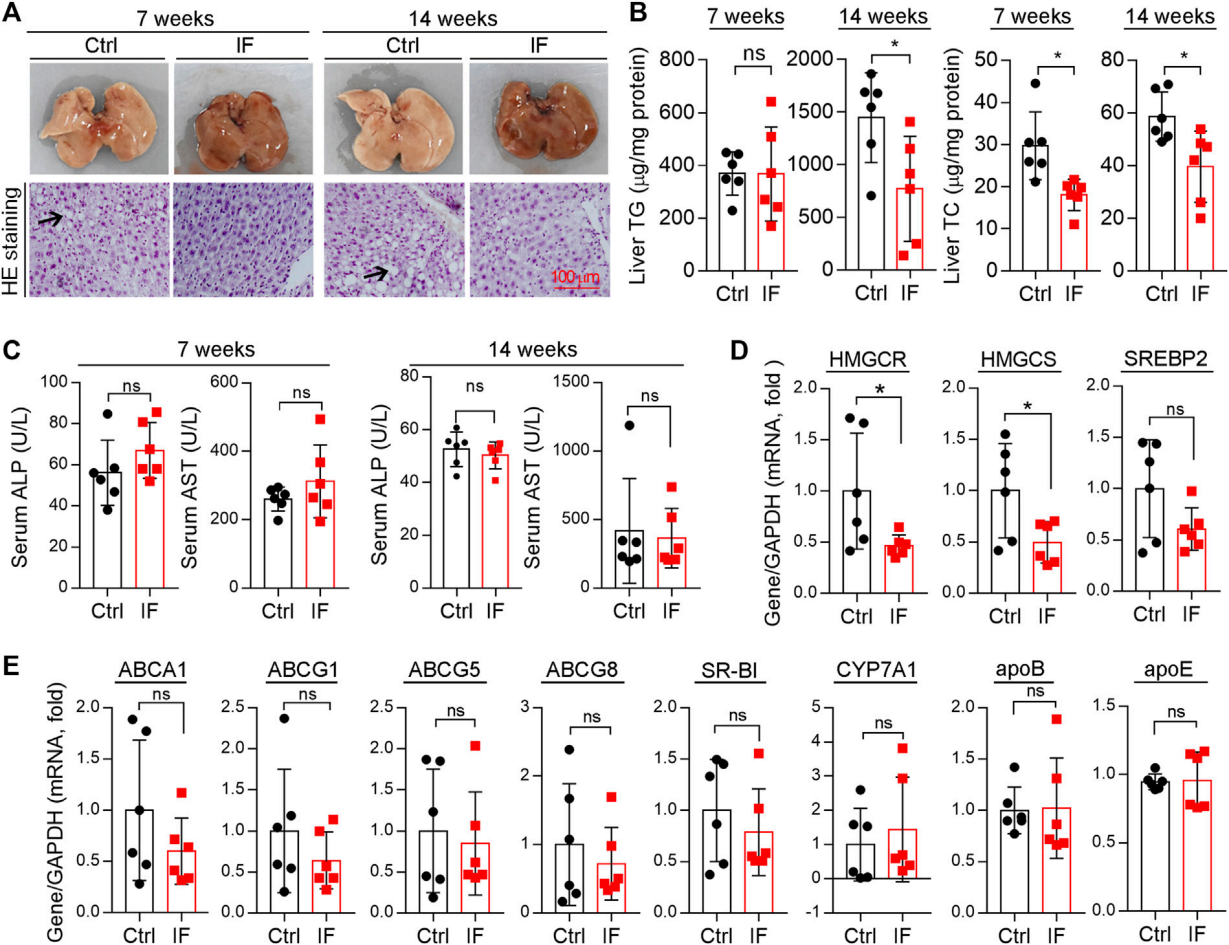
Intermittent fasting reduces HFD-induced lipid accumulation in the liver by reducing cholesterol synthesis. **(A)** Liver photos and HE staining of liver paraffin sections. Arrows indicate the vacuoles of lipid droplets after HE staining. **(B)**Liver TG and TC contents were quantitatively determined and normalized to protein content. **(C)**: serum ALP and AST levels were determined using the enzymatic kits. **(D,E)** Expression of genes related to cholesterol synthesis [**(D)**: HMGCR, HMGCS, and SREBP2], transport, and metabolism [**(E)**: ABCA1, ABCG1, ABCG5, ABCG8, SR-BI, CYP7A1, apoB, and apoE] were determined by qRT-PCR. *p < 0.05, ns: not significant (*n* = 6).

The liver is the main tissue for cholesterol synthesis/metabolism. Around 70–80% cholesterol is synthesized by the liver (Hotta and Chaikoff, 1955). Increased cholesterol synthesis in the liver can cause hypercholesterolemia, the main contributor to atherosclerosis. As shown in Figure 5A, the liver color turned pale and many vacuoles formed from lipid droplets after HE staining were seen in mice with continuous HFD feeding, particularly after 14 weeks of HFD feeding, suggesting the marked lipid accumulation in the liver. However, either liver color or morphology appears normal in IF groups. Quantitative analysis shows that the liver TG levels were substantially increased by the longer HFD feeding (∼3.5-fold, 7 week HFD *vs*. 14 week HFD), and the increase was potently blocked by IF (∼50%, left panels of Figure 5B). Interestingly, liver cholesterol levels were also significantly reduced by IF regardless of the time of treatment (right panel, Figure 5B). Furthermore, we determined that serum ALP and AST levels, two indicators for liver functions, were comparable between the control and IF groups (Figure 5C).

Associated with reduced cholesterol levels in circulation (Figure 4), total cholesterol levels in the liver were also reduced by IF (Figure 5B), indicating that the IF may regulate cholesterol synthesis/metabolism in the tissue. To uncover the mechanisms, we determined changes in genes responsible for cholesterol synthesis, transport, and metabolism in the liver. Compared with continuous HFD feeding, the feeding with the IF reduced expression of HMGCR and HMGCS, particularly HMGCR, the rate-limited enzyme in cholesterol synthesis (Figure 5D). The key transcriptional factor controlling HMGCR expression, SREBP2, was also slightly reduced (Figure 5D). However, expression of the genes involved in cholesterol transport (ABCA1, ABCG1/5/8, and SR-BI), cholesterol metabolism (CYP7A1), and apolipoprotein B/E (apoB and apoE) in LDLR^-/-^ mice were not affected by IF (Figure 5E). Therefore, the data in Figures 4, 5 suggest that IF ameliorated hypercholesterolemia is mainly attributed to inhibition of cholesterol synthesis in the liver.

### Intermittent Fasting Suppresses CCL2 Expression

**FIGURE 6:**
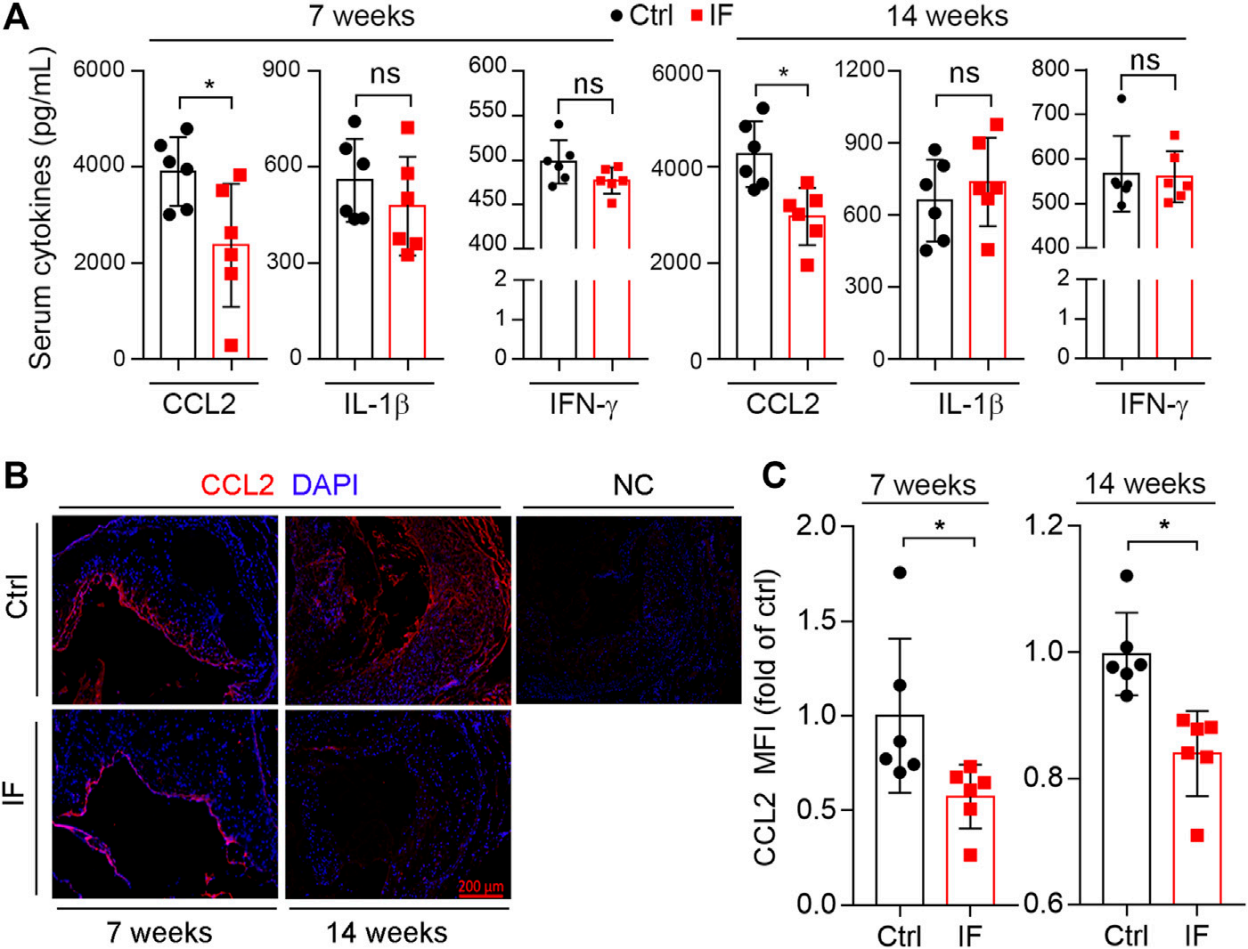
Intermittent fasting suppresses CCL2 expression. **(A)**: mouse serum CCL2, IL-1β, and IFN-γ levels were determined by the ELISA kits. **(B,C)**: aortic root cross sections were used to determine CCL2 expression by immunofluorescent staining with the quantification of CCL2-positive area **(C)**. *p < 0.05, ns: not significant (*n* = 6).

Accumulating studies have demonstrated that atherosclerosis is also a chronic inflammatory disease. Surprisingly, we determined that serum pro-inflammatory cytokines, IL-1β and IFN-γ, were not affected by IF. However, serum CCL2 levels were significant reduced by IF for either 7- or 14-week treatment (Figure 6A). CCL2 is a chemokine that attracts monocytes to the target site. Therefore, we determined CCL2 expression in plaques *via* immunofluorescence staining. Consistent with reduced CCL2 in serum, CCL2 expression was also significantly reduced by IF in lesions (Figures 6B,C).

### Intermittent Fasting Reduces Circulating Monocytes and the Adhesion to Endothelium

**FIGURE 7:**
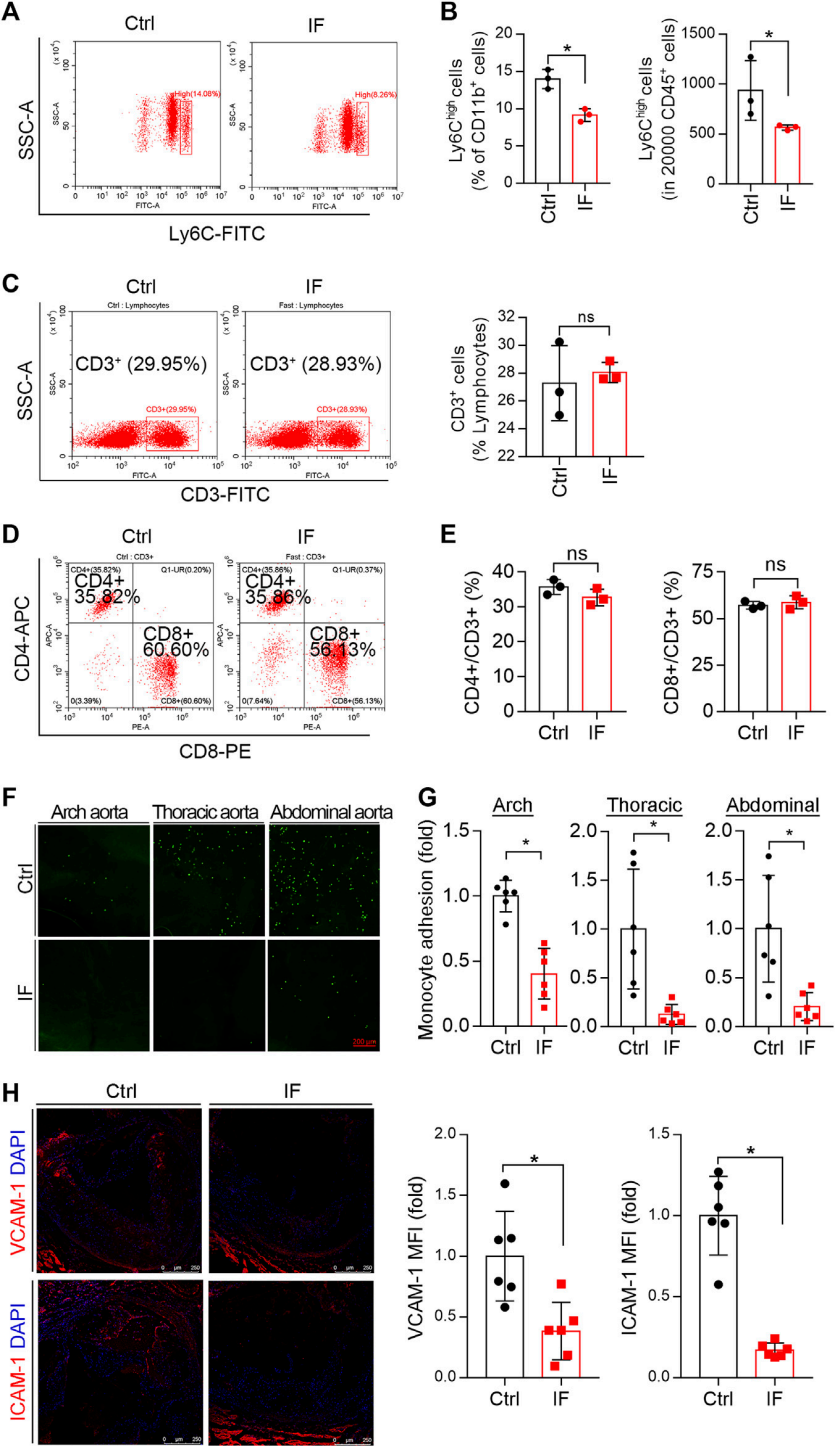
Intermittent fasting reduces circulating monocytes and their adhesion to endothelium. LDLR−/− mice were divided into the control or IF groups (*n* = 6/group). After 2 weeks of HFD feeding ad libitum or IF treatment, mouse peripheral blood and aorta samples were collected. Monocytes and T cells in blood samples were determined by FACS. **(A–B)**: Inflammatory monocytes (CD11b + CD45 + Ly6Chigh) were determined and quantified in total CD11b + or CD45 ^+^ cells. **(C–E)** CD3^+^ (total T cells), CD4^+^ T cells, and CD8^+^ T cells. *p < 0.05, ns: not significant (*n* = 3). **(F,G)** Arch aorta, thoracic aorta, and abdominal aorta were dissected from the collected aortas. Each of them were then cultured in complete DMEM medium containing CFSE-labeled THP-1 cells for 1 h. The adhering THP-1 cells were then captured by a microscope and quantitively analyzed. *p < 0.05 (*n* = 6). **(H)** The aortic root cross sections from mice in Figure 1 was used to determine the expression of VCAM-1 and ICAM-1 in plaques by immunofluorescent staining with quantification of VCAM-1 and ICAM-1–positive areas. *p < 0.05 (*n* = 6).

Considering decreased CCL2 levels in serum and plaques and CD68-positive cells in lesions, we speculated that IF might affect circulating monocyte distribution and its adhesion to endothelium. Therefore, we conducted IF treatment on LDLR^-/-^ mice for 2 weeks. At the end of the experiment, we collected mouse blood samples to determine the population of immune cells. The Ly6C^high^ cells were significantly reduced by IF (Figures 7A,B), indicating the treatment can reduce pro-inflammatory monocytes.

In addition to monocytes, T cells can also contribute to the development of atherosclerosis. However, we found CD3^+^ T cells were not significantly affected by IF (Figure 7C), while CD4^+^ nor CD8^+^ T cells were not changed either (Figure 7D).

At the initial stage of atherosclerosis, accumulated LDL in endothelium stimulates ECs to express/release adhesion factors and chemokines, which can attract circulating monocytes to adhere and migrate to the underneath of intima, where they can differentiate into macrophages. The reduced Ly6C^high^ cells by IF treatment (Figures 7A,B) suggests the adhesion of pro-inflammatory monocytes may also be reduced. To determine this, we collected mouse arch aorta, thoracic aorta, and abdominal aorta after 2-week IF to evaluate their monocyte adhesion capacity. As shown in Figure 7G, compared with continuous HFD feeding, the adhesion of CFSE-labeled THP-1 monocytic cells to different segments of the aorta was significantly reduced by IF treatment (Figures 7F,G). The adhesion of monocytes to endothelium was mediated by adhesion factors. Indeed, the expression of VCAM-1 and ICAM-1 was reduced by IF in the atherosclerotic plaques (Figure 7H). Taken together, Figure 7 shows that IF can reduce circulating pro-inflammatory monocytes and adhesion of these cells to endothelium, another anti-atherogenic mechanisms of IF treatment.

## Discussion

The proper fasting has been demonstrated to maintain fitness and decrease the risk factors of CVD and diabetes (Varady and Hellerstein, 2007; Andersen and Fernandez, 2013; Chen et al., 2016). However, the limited studies determined the effect of IF on CVD with mixed results. Some studies indicate IF can reduce the risk factors for CVD, such as serum lipid profiles and inflammatory cytokines. We previously reported that ∼40% calorie restriction inhibited atherosclerosis *via* attenuating body weight gain, improving lipid profiles, and inhibiting the expression of inflammatory molecules in lesions (Yang et al., 2020). In current study, we found that IF treatment significantly reduced lesions in the *en face* aorta or aortic root. In addition, it enhanced plaque stability by reducing necrotic cores and macrophage accumulation and increasing fibrotic cap thickness/collagen and SMC content. However, a previous study by Dorighello et al. (2014) demonstrated that alternate-day fasting induced obesity and diabetes, and worsened the development of spontaneous atherosclerosis in LDLR^-/-^ mice. The contradictory results with our study may be caused by methodological reasons in which Dorighello et al. fed the mice with normal chow, while we fed mice with HFD. The spontaneous development of atherosclerosis is slow, and the lesion was at an early asymptomatic stage. The early development of atherosclerosis is unable to speculate the clinical outcomes in the later stages. More time period should be applied with normal chow to clarify the exact effect of IF on spontaneous development of atherosclerosis.

Hypercholesterolemia is the main risk factor for CVDs. Thus, LDL management is the primary aim in clinical practice to reduce acute CVDs events. Statins, proprotein convertase subtilisin/kexin type 9 (PCSK9) inhibitors, apolipoprotein A-I–based therapies, and other lipid-lowering therapies have been demonstrated to reduce CVDs events (Zhang et al., 2016; Pulipati and Davidson, 2020). In our study, both TC and LDL-C were significantly decreased by IF treatment, indicating amelioration of hypercholesterolemia is one of the main contributors in the reduction of atherosclerosis. Serum cholesterol levels are affected by dietary intake, cholesterol synthesis, transport, and metabolism. We determined that the food intake was reduced by IF, suggesting the cholesterol intake is also reduced. Moreover, we observed that cholesterol levels in the liver were reduced by IF treatment, which should be attributed to reduced cholesterol synthesis. In addition, hepatic TG levels were also reduced by IF treatment. Reduced cholesterol and TG levels in the liver result in the decrease of lipid accumulation in the tissue and contribute to reduced atherosclerosis. However, the main transcriptional factor, SREBP2, which controls the biosynthesis of cholesterol in the liver, was not significantly affected by IF. Lu et al. demonstrated the post-prandial increase in insulin and the glucose concentration stimulates mTORC1 to phosphorylate the deubiquitylase ubiquitin-specific peptidase 20 (USP20) at S132 and S134, which is recruited to the HMGCR complex and antagonizes its degradation (Lu et al., 2020). Further studies should be done to determine if the reduced HMGCR expression by intermittent fasting is related with the mTORC1/USP20/HMGCR pathway.

LDL-C lowering treatment is insufficient to reduce CVD events in all patients. Indeed, atherosclerosis has also been recognized as a chronic inflammatory disease. Thus, the vascular inflammation, such as NLR family pyrin domain containing 3 (NLRP3) inflammasome-IL-1β pathway, can be another target for CVD treatment (Grebe et al., 2018). For instance, the results of the Canakinumab Anti-Inflammatory Thrombosis Outcomes Study demonstrate that treatment with monoclonal IL-1β-neutralizing antibody canakinumab reduces the risk of recurrent cardiovascular events in patients with prior heart attack (Ridker et al., 2017). However, in this study, we observed that IF had no effect on serum IL-1β levels, indicating IF-reduced atherosclerosis is unrelated to the regulation of inflammasome activity. IFNγ is another pro-inflammatory cytokine and mainly produced by T lymphocytes. It is involved in the innate and adaptive immune responses (Voloshyna et al., 2014), many of which are pro-atherogenic. IFNγ administration exacerbated AS *via* increasing the T-cell number within lesions in atherosclerosis-prone apoE^-/-^ mice (Whitman et al., 2000). Deletion of IFNγ reduces atherosclerosis in a sex-dependent manner in the rodent model (Whitman et al., 2002). We found that IF has no effect on IFNγ levels either, which is consistent with unchanged T cell population by IF treatment. Taken together, these results suggest that the IF treatment in our study has weak anti-inflammatory effects.

During atherosclerosis initiation/progression and under the influence of inflammatory stimuli, the splenic monocytes are recruited to the atherogenic vessel wall *via* the systemic circulation, where they adhere and enter the arterial vessel wall through the activated endothelial cells *via* binding to the specific adhesion molecules, such as VCAM-1, ICAM-1, P-selectin, and E-selectin (Fayad et al., 2018). In spite of the weak anti-inflammatory effect, we still observed that IF treatment clearly reduces monocyte adhesion or the migration of endothelium or the accumulation of macrophage/foam cells in plaques by reducing CCL2, VCAM-1, and ICAM-1 levels, and circulating inflammatory Ly6C^high^ monocytes. However, the exact mechanism of IF on Ly6C^high^ monocyte mobilization, CCL2 production was unclear. Pyruvate dehydrogenase kinase 2/4 (PDK2/4) and pyruvate dehydrogenase complex (PDC) were reported to be involved in glucose metabolism and insulin secretion during fasting (Sugden et al., 2001). PDK2/4 were also metabolic checkpoints for the polarization of macrophages to the M1 phenotype since PDK2/4 double knockout ameliorated inflammation (Min et al., 2019). We determined that the CCL2 (also known as MCP1) was reduced by IF, suggesting that the M1 phenotype polarization was reduced, which may be related with PDK2/4 and PDC expression. Moreover, it has been reported that alternate-day fasting protected cardiovascular system by activating autophagy (Godar et al., 2015). Autophagy plays an inverse function in cardiovascular disease, which can be triggered by ER stress (Kolattukudy and Niu, 2012). CCL2 was reported to induce plaque destabilization by increasing the activity of the ubiquitin–proteosome system in the inflammatory macrophages, which suggest that CCL2 may link ER stress to inflammation (Marfella et al., 2006). Considering the reduced CCL2 and necrotic core level by IF, it will be worthy investigating if autophagy involved in IF-reduced atherosclerosis in the future.

Taken together, our study for the first time demonstrates that IF treatment alleviates the development of atherosclerosis and increases plaque stability. Mechanistically, IF inhibits monocyte adhesion and infiltration to endothelium by reducing and circulating CCL2 levels and Ly6C^high^ monocytes. It also substantially reduces HFD-induced hypercholesterolemia by reducing the intake of cholesterol and cholesterol synthesis in the liver. Thus, our study also suggests that circulating monocytes and CCL2 levels should be considered as the key primary end points as other commonly determined circulating pro-inflammatory cytokines, such as c-reactive protein, IL-1β, or tumor necrosis factor. In conclusion, our study suggests IF can be an effective non-pharmaceutical therapy for atherosclerosis treatment and have potential clinical applications.

## Data Availability

The original contributions presented in the study are included in the article/Supplementary Material, and further inquiries can be directed to the corresponding author. The datasets used and/or analyzed during the current study are available from the corresponding author upon reasonable request.

## Abbreviations

ABCA1, ATP-binding cassette subfamily A member 1; ABCG1/5/8, ATP-binding cassette subfamily G member 1/5/8; ApoB, apolipoprotein B; CCL2, C-C motif chemokine ligand 2; CYP7A1, cytochrome p450 family 7 subfamily A member 1; HFD, high-fat diet; HMGCR, 3-hydroxy-3-methylglutaryl-CoA reductase; HMGCS: hydroxymethylglutaryl coenzyme A synthase; ICAM-1, intercellular adhesion molecule 1; IF, intermittent fasting; IFN-γ: interferon gamma; IL-1β, interleukin 1beta; LDL, low-density lipoprotein; MMP2/9: matrix metallopeptidase 2/9; SR-BI: scavenger receptor class B type I; SREBP2: sterol regulatory element binding protein 2; VCAM-1, vascular cell adhesion molecule 1.
